# Chimeric Antigen Receptor T-cell Therapy in the Treatment of Pediatric Acute Lymphoblastic Leukemia: Efficacy, Safety, and Future Directions

**DOI:** 10.7759/cureus.89172

**Published:** 2025-07-31

**Authors:** Hina Shamim, Kiran Jhakri, Moath Al-Shudifat, Bushra Sumra, Cyril Kocherry, Iana Malasevskaia

**Affiliations:** 1 Pediatrics, Baqai Medical University, Karachi, PAK; 2 Internal Medicine, Shahjalal University of Science and Technology, Sylhet, BGD; 3 Internal Medicine, Faculty of Medicine, Cairo University, Cairo, EGY; 4 Clinical Research, Sanmora Bespoke Clinical Research Solutions, East Orange, USA; 5 School of Medicine, Ninewells Hospital, Dundee, GBR; 6 Obstetrics and Gynecology, Private Clinic 'Yana Alexandr', Sana'a, YEM

**Keywords:** car-t cell therapy, complete remission, cytokine release syndrome, overall survival, pediatric acute lymphoblastic leukemia, pediatric oncology

## Abstract

Pediatric acute lymphoblastic leukemia (ALL) poses significant treatment challenges, particularly in relapsed or refractory cases. This review synthesizes recent studies evaluating the safety and efficacy of chimeric antigen receptor T (CAR-T) cell therapy, specifically tisagenlecleucel, in achieving complete remission (CR) and improving overall survival rates among pediatric patients with ALL. A comprehensive literature search identified 12 studies published between January 2014 and July 2024, encompassing cohort studies and clinical trials. Findings indicate that CAR-T cell therapy demonstrates superior CR rates (up to 100% in some studies) and manageable safety profiles, with common adverse effects, including cytokine release syndrome (CRS) and neurotoxicity. This review points out some of the important aspects such as the identification of biomarkers for response prediction, understanding of the mechanisms of resistance, and the crucial requirement for long-term outcome data. Challenges remain in the management of adverse effects, particularly CRS and neurotoxicity. This review underscores the transformative potential of CAR-T cell therapy in pediatric oncology while emphasizing critical areas for further investigation to optimize patient outcomes and enhance the therapeutic landscape for pediatric ALL.

## Introduction and background

Acute lymphoblastic leukemia (ALL) is a malignant hematological condition characterized by the uncontrolled proliferation of immature lymphoid cells in the bone marrow, blood, and other tissues [1].

Pediatric ALL poses a significant challenge within the field of pediatric oncology, accounting for approximately 25% of all childhood cancers [1]. 

ALL occurs in both children and adults, with its highest prevalence observed between the ages of two and five years [2]. Fortunately, children with ALL generally have a favorable prognosis compared to adults [3].

The incidence as per race and ethnic group is as follows: 14.8 cases per million black people, 35.6 cases per million white people, and 40.9 cases per million Hispanics. Childhood ALL develops more frequently in boys than girls (male: female ratio, 55% to 45%) [4].

**Figure 1:**
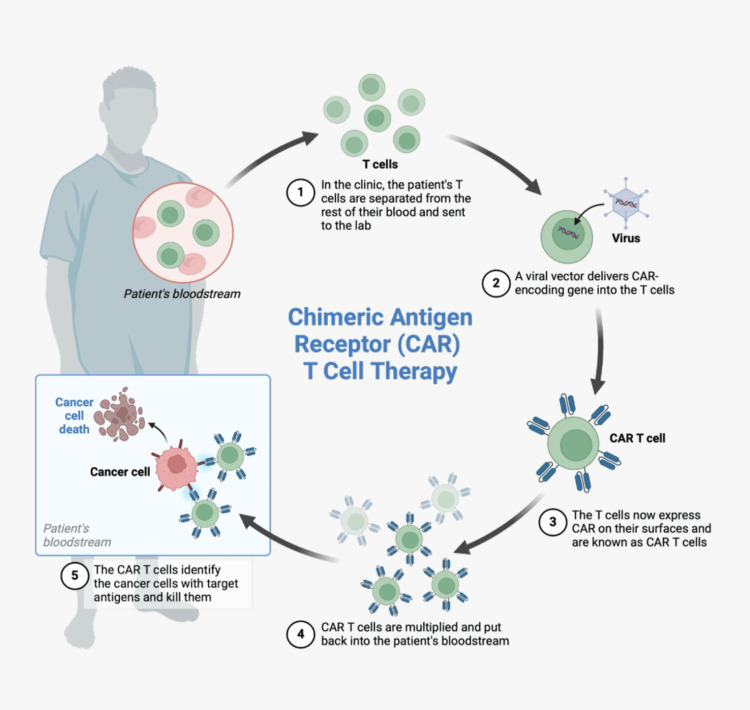
Chimeric antigen receptor therapy Figure created on Biorender

The first-line treatment for pediatric ALL is carried out in multiple phases, including induction, consolidation, intensification (which may involve reinduction or delayed intensification in certain protocols), and maintenance therapy to sustain remission [5]. Current treatment modalities for ALL include chemotherapy, radiation therapy, chemotherapy combined with stem cell transplantation, and targeted therapies, such as monoclonal antibodies [6]. Relapsed ALL is challenging to treat, but chimeric antigen receptor-modified T cells targeting CD19 offer a potential solution by overcoming the limitations of conventional therapies and inducing remission in patients with refractory disease [7]. This method involves the genetic engineering of a patient’s T-cells to target antigens present in cancer cells, hence amplifying the immune system's ability to recognize and eliminate malignant cells [8]. The process is illustrated well in Figure 1. 

The United States Food and Drug Administration's (FDA) approval of tisagenlecleucel in 2017 marked a significant milestone in the application of chimeric antigen receptor T (CAR-T) cell therapy for pediatric ALL [9]. This therapy has demonstrated considerable efficacy in achieving complete remission and improving survival rates among pediatric patients who have exhausted conventional treatment options [10]. Despite its promise, the safety profile and long-term implications of CAR-T cell therapy warrant further investigation [11]. The two adverse events that need to be addressed are cytokine release syndrome (CRS) and neurological events [12]. 

This literature review targets to synthesize recent research and clinical findings to evaluate the safety, efficacy, and challenges associated with CAR-T cell therapy in pediatric ALL. It highlights the potential of CAR-T cell therapy as a transformative therapeutic approach in the treatment of this condition. 

## Review

This literature review systematically evaluated the safety and effectiveness of CAR-T cell therapy in pediatric patients with ALL. Eligible studies included randomized controlled trials (RCTs), clinical trials, cohort studies, and case-control studies published in English between January 1, 2014, and July 31, 2024.

A comprehensive search strategy using keywords like "CAR-T cell therapy," "pediatric ALL," and ‘’complete remission" was conducted across PubMed, Medline, Science Direct, Cochrane Library (CENTRAL), and Europe PubMed Central (PMC). After removing duplicates and applying inclusion criteria, 12 articles were selected for detailed analysis.

Data extraction focused on study characteristics, sample sizes, interventions, outcomes, key findings, and safety profiles. A narrative synthesis provided a comprehensive overview of the results. The details of the included studies are presented in Table 1.

**Table 1:** Summary of included studies HSCT: Hematopoietic stem cell transplantation; B-ALL: B-cell acute lymphoblastic leukemia; AUTO 3: Autologous CAR-T product; CAR-T-allo-HSCT: Chimeric antigen receptor T-cell therapy followed by allogeneic hematopoietic stem cell transplantation; CNCT19: Inaticabtagene autoleucel; CR: Complete remission; CRi: Incomplete hematological remission; MRD: Minimal residual disease; CIR: Cumulative incidence of relapse; aGVHD: Acute graft-versus-host disease; cGVHD: Chronic graft-versus-host disease; NRM: Non-relapse mortality; LFS: Leukemia-free survival; OS: Overall survival; R/R: Relapsed/Refractory; EFS: Event free survival; CIBMTR CT Registry: Center for International Blood and Marrow Transplant Research Cellular Therapy Registry; ICANS: Immune effector cell-associated neurotoxicity syndrome

Author/Year	Study Design	Sample Size	Population	Intervention	Key Findings	Safety Profile
Maude et al., 2015 [13]	Phase 1 clinical trial	50 (with 14 receiving repeat infusion)	Children with relapsed/refractory ALL	Humanized and murine CD19-directed CAR T cells (CTL019 and CTL119)	Repeat infusion of murine CTL019 may prolong B cell aplasia; humanized CAR T cells showed efficacy in patients resistant to murine CAR T therapy.	Minimal toxicities (fever, fatigue); CRS in two patients, limited to mild symptoms; some patients experienced relapse.
Zhao et al., 2021 [14]	Prospective cohort study	105	2-52 years, with B-ALL	CAR-T-allo-HSCT Vs Chemotherapy-allo-HSCT	Second CR: The CAR-T group had a higher rate (78%) compared to the chemotherapy group (37%; p < 0.01). Complex Cytogenetics: more prevalent in the CAR-T group (44%) versus the chemotherapy group (6%; p < 0.001). Pre-transplant MRD: Similar proportions in both groups. Efficacy Measures at 4 Years Post-Transplant: CIR: 11.1% (CAR-T) vs. 12.8% (chemotherapy; p = 0.84). Cumulative Incidence of NRM: 18.7% vs. 23.1% (p = 0.641). LFS: 70.2% vs. 64.1% (p = 0.63). OS: 70.2% vs. 65.4% (p = 0.681).	Grade II-IV aGVHD: Higher in the CAR-T group (48.1%) compared to the chemotherapy group (25.6%; p = 0.016). Grade III-IV aGVHD: Similar incidence in both groups (11.1% for CAR-T vs. 11.5% for chemotherapy; p = 0.945). cGVHD: overall incidence was higher in the CAR-T group (73.3%) compared to the chemotherapy group (55.0%; p = 0.107). The rate of extensive cGVHD was similar (11.1% for CAR-T vs. 11.9% for chemotherapy; p = 0.964).
Curran et al., 2019 [15]	Multicenter clinical trial	25	1-22.5 years (pediatric/young adult) with R/R B-ALL	CD19-specific CAR T-cell therapy with conditioning chemotherapy (HD-Cy for 17 patients, LD-Cy for 8 patients)	Response and peak CAR T-cell expansion were superior in HD-Cy/MRD cohorts compared to LD-Cy/morphologic cohorts; all toxicities were reversible.	Severe CRS in 16% (4/25) and severe neurotoxicity in 28% (7/25); no increase in toxicity with higher dose intensity of conditioning chemotherapy.
Gu et al., 2020 [16]	Pilot study, clinical trial	22 patients (20 treated with CNCT19)	Pediatric and adult patients with (R/R B-ALL)	New CD19 CAR T (HI19α-4-1BB-ζ CAR T, or CNCT19) targeting a different epitope of the CD19 antigen.	90% of the 20 patients treated with infusions of CNCT19 cells reached CR/CRi within 28 days. The intention-to-treat analysis showed an 82% CR/CRi rate. Extramedullary leukemia disappeared completely in two relapsed patients post-infusion. Median overall survival was 12.91 months, and median RFS was 6.93 months after a median follow-up of 10.09 months. A low percentage of CD8+ naïve T cells was associated with shorter RFS (p = 0.012).	The study assessed the safety and feasibility of CNCT19-based therapy, indicating a favorable safety profile.
Chang et al., 2016 [17]	Multicenter clinical trial	125	Patients with chemo-resistant, CD19-positive acute B-ALL	4SCAR19 CAR T cell therapy	Complete response rates: 91.3% for <50% BM blast count (69 pts) and 75.8% for ≥50% (33 pts). Median overall survival (OS): 485 days (CI: [387, NA]) for cohort 1 and 317 days (CI: [135, NA]) for cohort 2 (P=0.03).	73 of 102 patients experienced 0-1 grade CRS; low toxicity observed even in high BM blast patients (>80%). Most had grade 1 (30) or grade 2 (13) CRS
Shah et al., 2021 [18]	Phase 1 clinical trial	50	Median age: 13.5 years (range: 4.3-30.4 years). Children and young adults (CAYAs) with R/R B-ALL.	Autologous CD19.28ζ-CAR T cells, followed by consideration for alloHSCT for eligible patients.	62.0% achieved CR 90.3% of CR patients were MRD-negative. Higher CR with fludarabine/cyclophosphamide (P = .041). Median OS: 10.5 months. 75.0% of MRD-negative CR patients proceeded to alloHSCT; median OS post-alloHSCT: 70.2 months. 24-month relapse incidence post-alloHSCT: 9.5%; 5-year EFS: 61.9%.	CRS: Occurred in 70% of patients; 18% had grade 3-4 CRS. Neurotoxicity: 20% Cardiac Events: 1, cardiac arrest with full recovery; severity of CRS linked to disease burden; higher burden correlated with increased CRS severity.
Cordoba et al., 2021 [19]	Phase 1 clinical trial	15	4-16 years, pediatric and young adult patients with R/R B-ALL	AUTO3 - a dual CAR T cell treatment targeting both CD19 and CD22	Remission rate of 86% at one month. One-year overall survival rate of 60%, event-free survival rate of 32%. Relapses likely due to limited long-term persistence of AUTO3.	Favorable safety profile with no dose-limiting toxicities. Grade 3–5 toxicity seen in 60% of patients, primarily grade 1-2 CRS. No severe CRS or neurotoxicity reported, with mild cases experienced by some patients
Shang et al., 2024 [20]	Cohort	42	Pediatric patients with R/R B-ALL	Anti-CD19 CAR T-cell therapy without bridging transplantation	100% CR rate by day 28 4-year OS: 61.3% ± 8.5% 4-year EFS: 55.9% ± 7.9% MRD ≥1% linked to inferior OS (P = .033) and EFS (P = .014)	Grade ≥3 CRS: 26.8% Neurotoxicity: 23.8% MRD ≥1% associated with increased severity of CRS and neurotoxicity.
Grupp et al., 2019 [21]	Real-world observational study using data from the CIBMTR CT Registry	159	Pediatric and young adult patients with R/R B-ALL	Tisagenlecleucel (CD19-directed CAR T-cell therapy).	Best overall response rate (CR/CRi): 88% 6-month OS: 94% MRD-negative in all tested patients.	Grade 3 or higher CRS: 13.3%; ICANS: 8.6%; Clinically significant infections: 35.2% 30-day mortality: 2 patients (not attributed to therapy)
Lee et al., 2015 [22]	Phase I clinical trial	39	4 months to 25 years, children and young adults with ALL	CD19 CAR T-cell therapy with standard salvage regimens	Overall CR rate: 59% (61% in ALL), 81% CR in low burden; 46% in high burden. Median LFS: 17.7 months.	Grade 4 CRS in 5.6% of patients. No neurotoxicity reported. Early intervention algorithm reduced severe CRS incidence
Maude et al., 2018 [23]	Clinical trial	75	Children 2-17 years with (R/R) CD19+ ALL	Murine CTL019 Reinfusion: Repeat infusion of murine CD19-specific CAR-modified T cells for patients with poor persistence. Humanized CTL119: Infusion of humanized CD19-directed CAR T cells for patients with B cell recovery or CD19+ relapse after prior therapy	Of 50 patients in CR at one month post-CTL019 infusion, 14 received a repeat murine CTL019 infusion. Humanized CTL119 showed efficacy in two patients. Immune-mediated rejection may be a mechanism of resistance noted in patients previously resistant to murine CTL019. The study suggests that repeat infusion of murine CTL019 can prolong B cell aplasia in certain patients, while humanized CAR T cells (CTL119) show promise in overcoming immune-mediated rejection, potentially improving outcomes in a refractory population.	Grade 3 or higher CRS: 13.3%; ICANS: 8.6%; Clinically significant infections: 35.2% 30-day mortality: two patients (not attributed to therapy)
Lerman et al., 2021 [24]	Prospective Cohort	206	7-12 years, patients with R/R B-ALL	Anti-CD19 CAR T cell therapy (4-1BB-containing products: CTL019 or humanized CART19)	51% achieved CR; 49% achieved complete remission with CRi RFS at 36 months: CR 57% vs. CRi 46% (p=0.2165) OS at 36 months: CR 81% vs. CRi 63% (p=0.0081)	CRi patients had a higher incidence of Grade 3/4 CRS (38% vs. 6.7% in CR group); no significant safety concerns reported beyond CRS.

Discussion

This literature review comprises 12 studies that systematically evaluated the safety and efficacy of CAR-T cell therapy in pediatric patients with ALL. The studies included a variety of designs, such as clinical trials and cohort studies. The sample sizes of the included studies ranged from a minimum of 15 to a maximum of 206 participants. The interventions focused on various CAR-T cell therapies targeting CD19. Several studies investigated murine and humanized CD19-directed CAR T cells, while others examined novel CAR constructs such as CNCT19 and AUTO3, which target both CD19 and CD22.

Efficacy of CAR-T Cell Therapy in Pediatric ALL

The efficacy of CAR-T cell therapy in pediatric patients with relapsed or refractory ALL has been supported through a series of studies that highlight both the potential and diversity of treatment outcomes. Maude et al. (2015) conducted a pivotal phase 1 clinical trial with 50 children, demonstrating significant efficacy of both humanized and murine CD19-directed CAR T cells [13]. Their findings indicated that repeat infusions of murine CTL019 could prolong B-cell aplasia, while humanized CAR T cells proved effective for patients who previously demonstrated resistance to murine therapy [13]. This emphasizes the importance of treatment strategies individualized to a patient's history and response.

In contrast, Zhao et al. (2021) presented a broader perspective through a prospective cohort study, which demonstrated a second complete remission (CR) rate of 78% in the CAR-T group compared to only 37% in the chemotherapy cohort (p < 0.01) [14]. This difference signifies the superior efficacy of CAR-T cell therapy, especially in patients with complex cytogenetic profiles, supporting its clinical utility in challenging cases. Curran et al. (2019) highlighted the role of conditioning regimens in their multicenter clinical trial [15]. They reported superior responses in patients who received high-dose conditioning chemotherapy prior to CAR T-cell infusion, although 16% of participants were observed to have severe CRS [15]. This finding represents that refining pre-treatment regimens can also improve the effectiveness of CAR-T cell therapies, particularly in the pediatric demographic, where initial therapeutic strategies can influence treatment responses.

The introduction of novel CAR constructs has also shown promise in improving treatment outcomes. Gu et al. (2020) reported an impressive 90% CR rate within 28 days among patients treated with the innovative CD19 CAR T (CNCT 19) [16]. This suggests that advancements in CAR technology may lead to stronger response rates, providing hope for patients who have limited options due to previous treatment failures. Chang et al. (2016) supported the significance of disease burden in treatment efficacy, reporting CR rates of 91.3% for patients with less than 50% bone marrow blast count [17]. This correlation between disease burden and treatment success further emphasizes the importance of careful patient selection and vigilant monitoring throughout the treatment process.

In a study by Shah et al. (2021), the importance of minimal residual disease (MRD) status was emphasized, with 62% of patients achieving CR and a median overall survival (OS) of 10.5 months [18]. Notably, 90.3% of CR patients were MRD-negative, suggesting that achieving MRD negativity is crucial for improved long-term outcomes [18]. This finding reiterates the need for ongoing assessment of MRD as a key prognostic marker in CAR-T cell therapy. Moreover, Cordoba et al. (2021) introduced the dual CAR T-cell treatment AUTO3, which targets both CD19 and CD22, reporting an 86% remission rate [19]. This approach supports the potential for enhanced efficacy in patient groups with few therapeutic options using multi-targeted therapies.

Shang et al. (2024) reported compelling evidence of the rapid effectiveness of CAR-T cell therapy, achieving a 100% CR rate by day 28, alongside a four-year OS of 61.3% [20]. This rapid response accentuates the groundbreaking potential of CAR-T cell therapy in pediatric ALL, especially in cases where prompt intervention is critical.

Lastly, the real-world observational study by Grupp et al. (2019) demonstrated an overall response rate (CR/CRi) of 88% with tisagenlecleucel, further solidifying the efficacy of CAR-T cell therapy in broader clinical practice [21]. This study highlights the applicability of CAR-T cell therapy outside of controlled clinical trials, fortifying its role as a cornerstone in the treatment of pediatric ALL.

The aggregate findings of these studies illustrate that CAR-T cell therapy represents a significant advancement in the treatment of pediatric ALL, showcasing varying degrees of efficacy influenced by factors such as treatment history, disease burden, and the evolution of CAR constructs. The ongoing refinement of these therapies continues to hold promise for improving outcomes in this vulnerable patient population.

Safety Profiles and Adverse Effects of CAR-T Cell Therapy

The safety profiles of CAR-T cell therapy, particularly in pediatric patients with ALL, reveal a complex relationship between therapeutic efficacy and potential adverse effects. This review synthesizes findings from included studies to provide a comprehensive understanding of the safety profiles associated with CAR-T cell therapy.

The most frequently reported adverse effect of CAR-T cell therapy is CRS. It is characterized by a systemic inflammatory response due to the rapid proliferation of CAR-T cells and subsequent cytokine release. The incidence of CRS varies across studies, with severe manifestations reported in up to 70% of patients, as seen in the study by Shah et al. (2021) [18]. In this study, 18% of participants experienced grade 3-4 CRS, indicating a significant clinical concern [18]. Similarly, Curran et al. (2019) noted severe CRS in 16% of their cohort, accentuating the need for vigilant monitoring and mitigation strategies to attenuate this potentially life-threatening complication [15].

The severity of CRS appears to correlate with the disease burden prior to treatment. Shah et al. (2021) observed that higher tumor loads were associated with increased CRS severity, suggesting that pretreatment assessments of disease burden could assist in risk stratification and management protocols [18]. Algorithmic early intervention, as stated by Lee et al. (2015), have demonstrated efficacy in reducing the incidence of severe CRS, highlighting the importance of proactive measures in clinical settings [22].

Another critical safety concern in pediatric patients receiving CAR-T cell therapy is neurotoxicity. Curran et al. (2019) documented severe neurotoxicity in 28% of their patients where adverse effects can manifest as confusion, seizures, and other neurological impairments, significantly impacting the quality of life for affected individuals [15]. In contrast, Lee et al. (2015) reported no neurotoxicity in their cohort and stated that variability in outcomes is based on specific CAR constructs and patient populations [22]. The underlying mechanisms of neurotoxicity remain an area of active investigation, with potential links to the inflammatory environment induced by CAR-T cell activation. Understanding these mechanisms is crucial for developing targeted interventions to prevent or diminish neurotoxic effects.

In addition to CRS and neurotoxicity, other adverse events associated with CAR-T cell therapy include acute and chronic graft-versus-host disease (aGVHD and cGVHD), infections, and hematologic complications. Zhao et al. (2021) reported a higher incidence of grade II-IV aGVHD in the CAR-T cell group compared to traditional chemotherapy, with 48.1% of patients experiencing this complication [14]. While the overall incidence of cGVHD was also higher in the CAR-T cell cohort, the rates of extensive cGVHD were comparable between CAR-T cell and chemotherapy groups [14].

Moreover, the risk of clinically significant infections post-CAR-T cell therapy remains a concern, as highlighted by Grupp et al. (2019), where 35.2% of patients experienced clinically significant infections [21]. This shows the importance of strong infection prevention strategies, particularly with reference to immunosuppression following CAR-T cell infusion.

While the efficacy of CAR-T cell therapy in achieving CR is well-documented, the associated risks necessitate careful patient selection, rigorous monitoring, and the implementation of proactive management strategies. Ongoing research is imperative to refine our understanding of the long-term safety implications of CAR-T cell therapy and to develop targeted interventions that can reduce adverse effects and maximize therapeutic benefits.

Strengths and Limitations of Included Studies and Review 

The review offers several strengths that enhance its value in assessing CAR-T cell therapy for pediatric ALL. By incorporating a range of studies, it provides a thorough perspective on the therapy's safety and efficacy, enabling a deeper understanding of its impact across various contexts and methodologies. A major strength is the thorough analysis of the therapy’s safety and efficacy, which is crucial for assessing its clinical application. By critically evaluating both positive outcomes and adverse effects, the review presents a refined view of CAR-T cell therapy.

However, the review also has notable limitations. While some studies offer extended follow-up, many focus primarily on short- to medium-term outcomes. Given the recent introduction of CAR-T cell therapy, comprehensive long-term data regarding durability, late side effects, and overall survival remain scarce. Moreover, several studies are constrained by small sample sizes or are conducted at single centers, which can affect the statistical power of the findings and may not fully represent the broader pediatric ALL population. Also while the review focuses on clinical outcomes, it does not provide a thorough assessment of the economic impact of CAR-T cell therapy, including costs, accessibility, and long-term financial sustainability. This could be a critical factor in assessing the feasibility of widespread clinical use.

Additionally, the differences in patient populations, treatment protocols, and outcome measures can complicate the ability to draw generalized conclusions. Inconsistent reporting of adverse effects, such as CRS and neurotoxicity, further complicates the assessment of the safety profile of CAR-T cell therapy. Variations in grading systems and definitions can conceal the accurate evaluation of these critical outcomes. Finally, the absence of a systematic review methodology and formal quality assessment of the included studies limits the ability to critically evaluate the strength of the evidence presented, potentially affecting the reliability of the conclusions drawn from the review.

Future Directions for Research

Future research on CAR-T cell therapy in pediatric ALL should prioritize longitudinal studies to assess the long-term durability of treatment responses and late-onset effects associated with this innovative therapy. Understanding long-term survival rates, quality of life, and potential late complications is essential for establishing the overall efficacy and safety profile of CAR-T cell therapy. Identifying biomarkers that predict patient responses to CAR-T cell therapy and the likelihood of adverse events, such as CRS and neurotoxicity, will enable personalized treatment plans and improve patient stratification.

Comparative effectiveness research is crucial; head-to-head trials comparing CAR-T cell therapy with emerging therapies, including bispecific T-cell engagers and novel immunotherapies, will provide insights into optimal treatment strategies for pediatric ALL. Investigating mechanisms of resistance to CAR-T cell therapy, particularly in patients who experience relapse after initial success, can inform the development of combination therapies or alternative strategies. Expanding research to include diverse populations and real-world settings will enhance the generalizability of findings, taking into account genetic, environmental, and socioeconomic factors that may influence treatment outcomes. Lastly, further studies are needed to refine protocols for managing adverse effects, particularly CRS and neurotoxicity, to minimize their impact on treatment success and patient quality of life.

Clinical Implications

The growing body of evidence supporting the efficacy of CAR-T cell therapy in achieving CR in pediatric and young adult ALL underscores the need for clinicians to consider this treatment option for patients with relapsed or refractory disease, particularly those who have not responded to conventional therapies. It is imperative that healthcare providers implement rigorous monitoring protocols for patients undergoing CAR-T cell therapy, including regular assessments for CRS and neurotoxicity severity. Early intervention strategies, as highlighted in recent studies, can significantly reduce the severity of these complications and improve patient outcomes. 

A multidisciplinary approach is essential, involving oncologists, immunologists, nursing staff, and supportive care specialists to ensure comprehensive management of both the therapeutic and adverse effects of CAR-T cell therapy. Collaborative care models can facilitate timely interventions and enhance the overall quality of care provided to these vulnerable patients.

## Conclusions

This review highlights the potential of CAR-T cell therapy in the transformation of pediatric ALL treatment, particularly in relapsed and refractory cases. The synthesis of recent studies demonstrates that CAR-T cell therapy, especially tisagenlecleucel, has achieved impressive CR rates and has improved overall survival, offering an alternative for patients who have exhausted conventional treatment options. However, the challenges of adverse effects such as CRS and neurotoxicity require careful monitoring and management.

Further research is required to address existing gaps in knowledge, particularly regarding long-term outcomes and the identification of predictive biomarkers. Understanding the mechanisms of resistance to CAR-T cell therapy is also important in developing more effective treatment strategies. A multidisciplinary approach to optimize patient care is essential to ensure that both the therapeutic benefits and potential risks are managed effectively. While CAR-T cell therapy represents a significant advancement in pediatric oncology, ongoing research and clinical vigilance are vital to maximize its benefits and reduce associated risks, ultimately enhancing the therapeutic landscape for children and young adults with ALL.
